# MAGIS syndrome: Phenotypes, pathogenesis, and treatment

**DOI:** 10.70962/jhi.20250065

**Published:** 2025-08-14

**Authors:** Ian T. Lamborn, Huie Jing, Eesha Chattopadhyay, Hyoungjun Ham, Yu Zhang, Helen C. Su

**Affiliations:** 1 Lymphocyte Biology Section, Laboratory of Immune System Biology, Intramural Research Program, National Institute of Allergy and Infectious Diseases (NIAID), National Institutes of Health (NIH), Bethesda, MD, USA; 2 Human Immunological Diseases Section, Laboratory of Clinical Immunology and Microbiology, Intramural Research Program, NIAID, NIH, Bethesda, MD, USA; 3 NIAID Clinical Genomics Program, Intramural Research Program, NIAID, NIH, Bethesda, MD, USA; 4 Department of Urology, Mayo Clinic, Rochester, MN, USA

## Abstract

Inborn errors of immunity (IEI) presenting with immunodeficiency and autoimmunity can illuminate pathways essential for immunocompetence and self-tolerance. We recently characterized a new IEI named MAGIS (“Midline malformations of the brain, Anterior pituitary gland dysfunction, Growth retardation, Immunodysregulation/Immunodeficiency, and Skeletal defects”) caused by heterozygous germline-activating mutations in *GNAI2* (encoding the heterotrimeric G protein, G_αi2_). This disorder demonstrates the central role of G_αi2_ regulation of chemotaxis in humans and a novel pathway by which G proteins regulate T cell activation. Here, we review the clinical features, current genetic and biochemical understanding, and future therapeutic considerations for this new syndromic immune dysregulation disorder.

## Teaser

The inherited syndromic immunodysregulatory disorder MAGIS demonstrates the central role of G_αi2_ regulation of chemotaxis in humans and a novel pathway by which G proteins regulate T cell activation. Here, the authors review MAGIS clinical features, current genetic and biochemical understanding, and future therapeutic considerations.

## Introduction

The study of inborn errors of immunity (IEI) remains among the best available tools for understanding how the immune system functions in humans. Increasingly, immune dysregulation disorders have been recognized as a unique subgroup of IEI that present with autoimmunity or autoinflammation, often in addition to immunodeficiency (1, 2). As such, immune dysregulation disorders offer insight into pathways regulating protection from both infectious diseases and the immune responses necessary to control them. We recently described a new syndromic immune dysregulation disorder (3), which we term MAGIS, a mnemonic chosen to capture its salient clinical features (described below). While the full biochemical, cellular, and clinical consequences of MAGIS remain to be uncovered, early investigation of MAGIS has delineated novel pathways of cross-talk between chemokine receptor signaling and T cell activation, providing insight into normal immune system biology as well as potential targets of therapeutic intervention (3). The goals of this review are to (1) highlight the clinical features of MAGIS so as to improve recognition and diagnosis of this newly described disorder, (2) summarize our current understanding of the genetic and biochemical underpinnings of MAGIS that contribute to disease features, and (3) discuss potential treatments on the horizon based upon this current understanding.

## Clinical features

**Figure 1.**
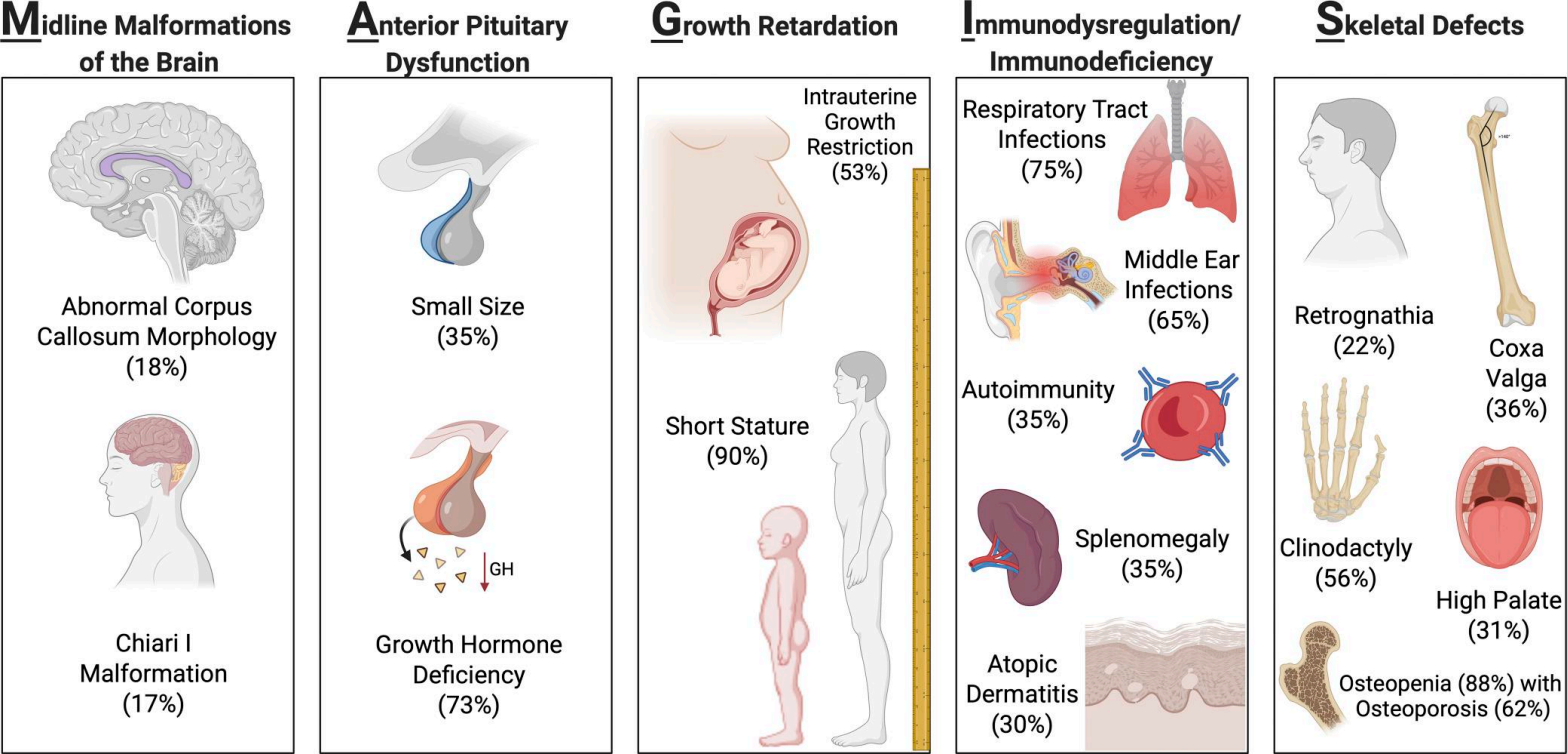
**Clinical features of MAGIS syndrome.** The acronym captures five cardinal features, namely, Midline malformations of the brain, Anterior pituitary gland dysfunction, Growth retardation, Immunodysregulation/immunodeficiency, and Skeletal defects. Percentages indicate frequencies in our patient cohort. The Latin word *magis* means “more,” which is helpful in remembering that the *GNAI2* mutations in this syndrome are gain-of-function (GOF) (more than WT).

We initially reported 20 individuals from 18 families with germline-activating mutations in *GNAI2*, encoding G_αi2_ (3). G_αi2_ is a heterotrimeric G protein that transduces signals from G protein–coupled receptors (GPCRs) in response to a wide variety of extracellular stimuli (4, 5, 6). It is expressed throughout the body but at high levels within the immune system. Consistent with the ubiquitous expression of G_αi2_ throughout development, patients harboring these mutations exhibit multisystem developmental abnormalities and organ dysfunction. This syndrome has now been named MAGIS, an acronym that denotes five cardinal syndromic features: Midline malformations of the brain, Anterior pituitary gland dysfunction, Growth retardation, Immunodysregulation/Immunodeficiency, and Skeletal defects (Fig. 1). Despite these core features, diagnosis can be challenging as MAGIS syndrome presents with considerable phenotypic heterogeneity. Broadly, nearly all patients display both immune and nonimmune disease features, but with variable severity.

Nonimmune abnormalities in MAGIS syndrome affect most organ systems including nervous, endocrine, respiratory, cardiovascular, gastrointestinal, dermatological, and skeletal systems in diverse ways (see Supplementary Text 3 and 4 in [3] for more detail). Among these, growth retardation is most penetrant (90%), manifesting as both prenatal intrauterine growth restriction (IUGR; 53%) and postnatal short stature (90%), as well as low serum insulin-like growth factor-1 (8 of 11 tested, 73%) and growth hormone deficiency (7 of 7 formally tested). Further endocrinological evaluation has revealed that structural (41%) or functional (53%) defects of the anterior pituitary gland are present in 59% of patients. Excluding pituitary and sella turcica defects, MAGIS patients also exhibit various midline brain malformations (30%) including agenesis or hypoplasia of the corpus callosum, cerebellar hypoplasia, pons hypoplasia, and Chiari I malformations among others. Skeletal defects comprise diverse craniofacial, appendicular, and axial skeletal dysostoses affecting 85% of patients and underlie the high prevalence of dysmorphia (100%). While these four central nonimmune features are captured in the MAGIS acronym, it is important to note that patients can also bear significant cardiovascular, pulmonary, gastrointestinal, genitourinary, and dermatological disease described elsewhere (3). While some of these other features, such as cryptorchidism or gut malrotation, are prevalent in the general population, the presence of extremely rare features, such as coloboma or subcortical band heterotopia (double cortex syndrome), should automatically raise suspicion for an underlying MAGIS diagnosis.

Immune-mediated disease (95%) is widely present in MAGIS as both immunodeficiency (90%) and immune dysregulation (50%; systemic autoinflammation, 15%, autoimmunity, 35%, and splenomegaly, 35%). In distinction from other, typically loss-of-function IEI, which predispose affected individuals to a narrow infectious phenotype or particular mode of exposure (7, 8), MAGIS appears to confer some susceptibility to a broad range of common and uncommon microbes. Sinopulmonary (respiratory tract, 75%; middle ear, 65%; and sinuses, 35%), cutaneous (25%), and invasive (meningitis, 10%; bacteremia, 20%; and cutaneous abscess, 15%) infections were the most prevalent sites of infection resulting from diverse families of bacteria and viruses. Notably, despite demonstration of a clear leukocyte chemotaxis defect (detailed below in the section “Biochemical impact of mutant G_αi2_ on immunity”), only 25% of our patient cohort was extensively affected by cutaneous viral infections, primarily from human papillomavirus–related warts. Immune dysregulation manifested sporadically in individual patients as life-threatening lymphocytic infiltration of the brain, lung, or liver, and macrophage activation syndrome, as well as more commonly autoimmunity (35%) and splenomegaly (35%). Autoimmune manifestations included life-threatening autoimmune hemolytic anemia (20%), autoimmune thrombocytopenia (10%), psoriasis (10%), and Hashimoto’s thyroiditis, type I diabetes mellitus, alopecia, celiac disease, and autoimmune enteritis in individual patients to date.

**Figure 2.**
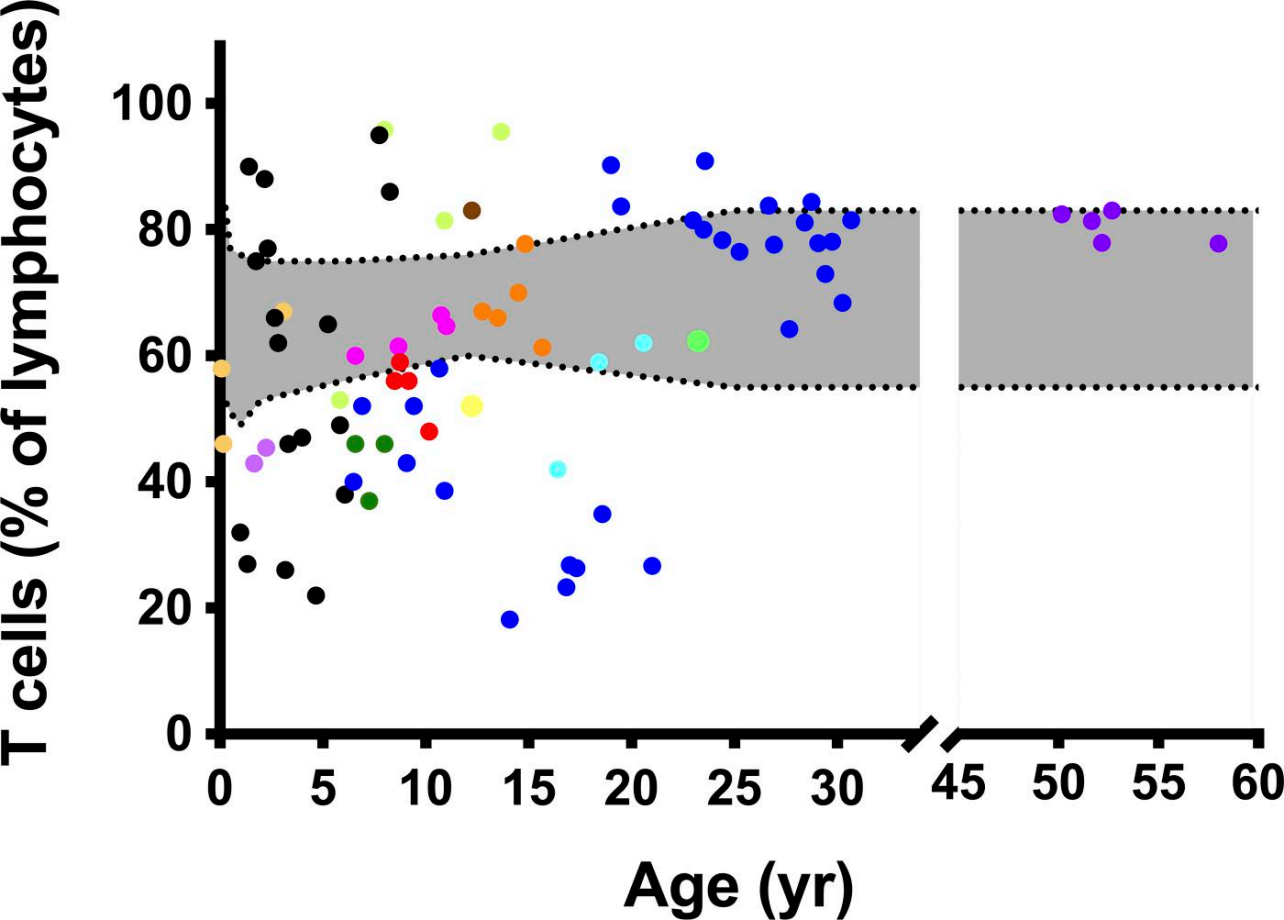
**Variable peripheral blood CD3** ^**+**^ **T cell counts in MAGIS patients over time.** Each different colored symbol corresponds to an individual MAGIS patient in our original cohort, with beige representing P13. The graph shows relative values; absolute values, which are usually low or normal, were previously published as Fig. S5 B in (3). Gray region indicates the reference ranges for healthy normal donors according to age.

Overall, given the potential for severe immune-mediated disease, MAGIS patients warrant full immunological workup, even if they initially present with nonimmune features (see Supplementary Text 5 in [3] for more detail). Such evaluation should include quantitative immunoglobulins and vaccine titers, with consideration for immunoglobulin replacement therapy if patients exhibit low total IgG, nonprotective vaccine responses, or persistent infectious burden suggestive of defective humoral immunity. Considerable inter- and sometimes even intraindividual variability is observed, as shown in the example for T cells depicted in Fig. 2 (see also Fig. S5 in [3] for other immune parameters). Complete blood counts with differentials over time reveal a trend of high/normal monocytes and neutrophils and low/normal lymphocytes. Newborn screen for severe combined immunodeficiency (SCID) was abnormal in one patient, who had an absolute T cell count of 0.577 × 10^3^ cells/µl at 7 wk of age increasing to 1.486 × 10^3^ cells/μl 1 mo later (see P13 in Supplementary Text 4, Fig. 2, and Fig. S5 in [3]). In general, lymphocyte immunophenotyping in peripheral blood demonstrates low/normal T cells often with improvement with age, low naïve-to-memory T cell ratio, and low B cells with associated low serum IgM. Additionally, mitogen- or antigen- induced proliferation studies are typically normal or increased, consistent with the T cell hyperresponsiveness phenotype linked to this disease (see also Fig. S6 in [3]). To the limited extent that they have been done, clinical tests of complement function, neutrophil function, and innate immune responses in MAGIS patients have been normal.

The phenotypic variability in MAGIS can be appreciated by reviewing intrafamilial cases in the original patient cohort. In family XIII, both the affected proband (P13) and his father (P14) carry the Arg179Cys mutation (see Supplementary Text 4 and Table S2 in [3]). P13 at 2 years of age had IUGR, congenital heart valve abnormalities with hydrops fetalis, dysmorphism (retrognathia, high forehead/anterior hairline, low-set ears), short femurs, hypospadias, severe congenital sensorineural hearing loss, neurodevelopmental delay, behavioral abnormalities, stereotypy, small pons and thin corpus callosum, feeding difficulties (constipation, gastroesophageal reflux), postnatal growth delay, and multiple neonatal infections including urosepsis with pan-lymphocytopenia. By contrast, his father has dysmorphism (midface retrusion, high forehead/anterior hairline, posteriorly rotated ears with other pinna abnormalities, upslanting palpebral fissures, finger clinodactyly), osteoporosis, cryptorchidism, hearing loss with vestibular dysfunction, neurodevelopmental delay with borderline intellectual disability, epilepsy, migraine headaches, anxiety, and a midline arachnoidal cyst. P14 also had growth delay with short stature, an annular pancreas with malrotation of the bowel and esophageal hiatal hernia, gastroesophageal reflux with aspiration pneumonias, recurrent infections (sinusitis, otitis media, tonsillitis, paronychia; lymphocyte numbers not measured), and celiac disease. Overall, while both patients share similar dysmorphic features, external genitalia defects, growth impairment, neurodevelopmental delays, and recurrent infections, some abnormalities were carried by only one individual, such as the heart valve defects in P13 or the anatomical gastrointestinal defects in P14.

Another illustrative example is family XVII, in which two affected brothers (P18, P19) carried the Arg179His mutation (see Supplementary Text 4 and Table S2 in [3]). P18 has dysmorphism (micrognathia, high anterior hairline, low-set ears), cryptorchidism, inguinal hernia, pelvic kidney, childhood failure to thrive with adult short stature, arthritis, gout, suspected osteoporosis, chronic diarrhea, sensorineural hearing loss, compensated hypothyroidism, anxiety, depression, asthma, recurrent infections (otitis media, croup, pharyngitis, bronchitis, pneumonia), and dysgammaglobulinemia (low serum IgM, poor vaccine titers). His deceased brother P19 had dysmorphism (micrognathia, high anterior hairline, low-set ears), cryptorchidism, inguinal hernia, deviated nasal septum, IUGR with childhood failure to thrive and adult short stature, neurodevelopmental delay, epilepsy, ataxia, nystagmus, migraine headaches, autism spectrum disorder, obsessive–compulsive disorder, anxiety, depression, psychosis, irritable bowel syndrome, mild splenomegaly, recurrent infections (otitis media, pneumonia, bronchitis, sinusitis, shingles), dysgammaglobulinemia (low serum IgM, low isohemagglutinins, and variably low vaccine titers), and T cell lymphopenia. Overall, both patients shared dysmorphism with cryptorchidism, inguinal hernia, chronic diarrhea, growth delay, anxiety and depression, and recurrent infections with dysgammaglobulinemia, but features only present in one individual include the arthritis in P18 or the neurodevelopmental delay and T cell lymphopenia in P19. In summary, in our limited cohort of 20 patients, MAGIS displayed broad clinical heterogeneity, which is observed even for relatives carrying the same *GNAI2* variant, and which may pose challenges for early diagnosis. While we observed a relatively low premature mortality (10% in our original cohort of 20 patients), disease involvement in multiple organ systems suggests potential for considerable morbidity particularly during embryonic/fetal development.

## Genetics

The 20 MAGIS patients reported so far represent multiple ancestries from around the world (3). Each patient carried an extremely rare or not previously reported heterozygous missense variant in *GNAI2*, with pathogenic predictions by several computational algorithms validated by extensive biochemical testing (see section below). All such patient variants are absent from general population genetic databases except for rs137853227 (Arg179His), which was found in two individuals in gnomAD v4.1.0, accessed March 2025 (minor allele frequency 0.000001281, one of African American ancestry and the other of European ancestry), having unknown affection status. MAGIS displayed full disease penetrance in the families we studied, with mutations recurring among unrelated patients often observed at residues Thr182 (Thr182Ala/Ile/Pro in six families) and Arg179 (Arg179His/Cys in seven patients from five families). Most pathogenic variants occur in a de novo pattern with mutations detected in several tissues, suggesting the de novo variants arose in germ cells or early during embryonic development (3). However, in our patient cohort one family had the Arg179Cys mutation in father and son, and another family had the Arg179His mutation in two brothers (described in the preceding section), consistent with an autosomal dominant inheritance pattern. These observations suggest that additional rare, unrecognized patients with subclinical disease likely exist in the general population.

## Biochemical impact on mutant G_αi2_ activity

**Figure 3.**
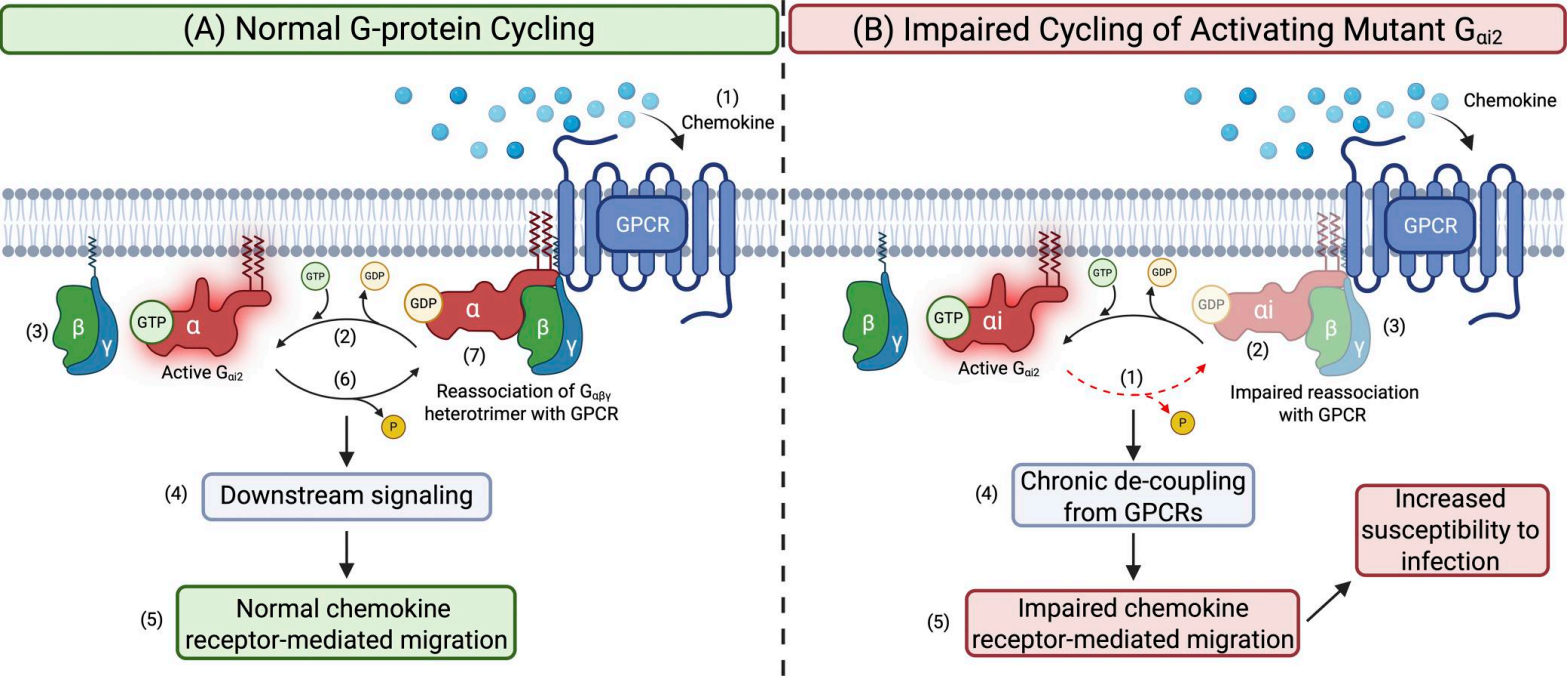
**Activating G**_**αi2**_ **mutations impair G protein cycling for GPCR signaling. (A)** Normal WT G_α_: GPCR ligation (1) activates heterotrimeric G proteins by exchanging GTP for GDP on the G_α_ subunit (2), causing G_α_ disassociation from the G_βγ_ heterodimer (3), and initiating downstream signaling (4) for cellular responses such as cell migration in the case of G_αi2_ (5). To terminate signaling, G_α_ uses its GTPase activity to hydrolyze GTP into GDP (6), allowing the inactivated G_α_ to reassemble into the G_αβγ_ heterotrimer and reassociate with a GPCR (7) for a new activation cycle. **(B)** Mutant G_α:_ activating mutations in G_αi2_ impair hydrolysis of G_αi2_-bound GTP (1), delaying conversion of G_αi2_ back into its inactive GDP-bound form (2) and hence G_αi2_ reassociation with the G_βγ_ heterodimer and GPCR (3). The chronic decoupling of the heterotrimeric G proteins from GPCRs (4) impairs responses to GPCR ligands such as chemokine receptor–mediated migration (5).

G_α__i2_ is an α inhibitory (G_αi_) subunit of heterotrimeric G protein (G_αβγ_) complexes, which propagate signals from GPCRs (Fig. 3 A) (4, 5, 6). GPCR ligation induces G_α_ to release bound GDP and bind GTP. The activated G_α_-GTP subunit then dissociates from the G_βγ_ complex and from GPCR, enabling both G_α_-GTP and G_βγ_ to initiate downstream signals. Eventually, G_α_ hydrolyzes GTP into GDP, terminating signaling and permitting reassembly of the inactive G_αβγ_ heterotrimer–GPCR complex (9). The G_αi2_ amino acid residues altered in MAGIS patients are highly conserved across species in other G_α_ (see Fig. S1 in [3]) and RAS superfamily members (10). The patients’ mutations were clustered in the P-loop and switch regions of the Ras-like domain of G_α_, which is critical for guanine nucleotide binding and GTPase activity. Biochemical analysis demonstrated that mutant G_αi2_ binds GTP up to 20-fold faster than wild-type (WT) and hydrolyzes GTP up to 100-fold slower than WT G_αi2_ (3). Furthermore, most mutant G_αi2_ proteins are insensitive to inactivation by regulators of G protein signaling proteins, a family of GTPase-activating proteins (GAPs) that normally accelerate GTPase activity of G_α_ protein (3, 11). Therefore, mutant G_αi2_ proteins in MAGIS patients are constitutively activated through multiple mechanisms: faster GTP binding, decreased GTPase activity, and GAP insensitivity.

It should be pointed out that for the intrafamilial cases discussed in the above “Clinical features” section, both the Arg179His and Arg179Cys mutant G_αi2_ proteins impair intrinsic GTP hydrolysis and hence are “activating” but less severely so than the other mutants. The Arg179His and Arg179Cys G_αi2_ proteins bind GTP more slowly than WT G_αi2_ and remain sensitive to the GTP hydrolysis–promoting effects of GAPs (see Table S2 and Fig. 1, D–G, in [3]). By contrast, other patients’ mutant G_αi2_ proteins bind GTP faster than WT G_αi2_ and are insensitive to the GTP hydrolysis–promoting effects of GAPs. These characteristics explain why the Arg179His/Cys variants can be passed on through successive generations and, while extremely rare, be found in the general population (see above in the “Genetics” section).

## Biochemical impact of mutant G_αi2_ on immunity

Heterotrimeric G proteins transduce signals intracellularly in response to a wide variety of extracellular stimuli, including hormones, neurotransmitters, and chemokines received from GPCRs (4, 5). G_αi2_ is a major mediator of chemokine signaling for migration of leukocytes (12, 13, 14, 15, 16, 17, 18, 19). Indeed, MAGIS patients’ T cells and neutrophils exhibit impaired in vitro and in vivo cellular migration, as well as impaired chemokine-induced calcium fluxes to multiple chemokines or other chemoattractants (3). Expressing mutant G_αi2_ proteins in healthy control primary T cells or cell lines is sufficient to recapitulate the patients’ cellular defects (3). Further investigation using a bioluminescence resonance energy transfer assay, a sensitive method for measuring the proximity of labeled proteins to one another, demonstrated that MAGIS mutant proteins remain predominantly decoupled from GPCRs at steady state (3), consistent with their biochemically activated state. As such, mutant proteins are minimally responsive to chemokine receptor ligation and unable to integrate chemotaxis signals accurately (Fig. 3 B). Together, these findings support a chemotaxis defect affecting both myeloid and lymphoid compartments to a broad range of chemokines, a cellular finding that is consistent with other immunodeficiencies to mucocutaneous bacterial and viral infections (20, 21, 22, 23). These findings also predict that patients with complete G_αi2_ deficiency, as yet unidentified, will share a common mechanism of impaired chemotaxis due to compromised chemokine receptor signal transduction, resulting in increased infection susceptibility. The ubiquitous expression pattern of G_αi2_ and nonimmune birth defects seen in MAGIS patients also suggest that chemotaxis of nonhematopoietic cells may also be affected during embryonic/fetal development.

One IEI in particular with some parallels to MAGIS is WHIM (Warts, Hypogammaglobulinemia, Infections, and Myelokathexis) syndrome caused by heterozygous GOF mutations in the C-terminal end of the G_αi2_-dependent chemokine receptor CXCR4. Clinically, MAGIS and WHIM syndromes share some features, including intermittently present nonimmune features such as congenital heart and cerebellar birth defects (24, 25, 26, 27, 28). However, a closer look reveals clinical distinctions, which highlight the two different molecular mechanisms of disease. Like MAGIS, WHIM is characterized by recurrent bacterial and viral mucocutaneous infections including, most notably, human papillomavirus–driven warts—a feature seen in a significant minority (25%) of known MAGIS patients, albeit to a less severe degree than has been described for WHIM syndrome (26, 29, 30). Hypogammaglobulinemia is also present in both diseases, affecting 20% of known MAGIS patients compared with 58–89% in WHIM syndrome (31, 32). One hallmark of WHIM syndrome, myelokathexis, has not been observed in any patients with MAGIS with the caveat that bone marrow examinations have been limited (three patients to date), and neutropenia, which is associated with myelokathexis in WHIM syndrome, was only intermittently seen in one critically ill MAGIS patient. The disparity highlights the distinct mechanistic underpinnings of these two syndromes. While WHIM mutations impair CXCR4 downregulation resulting in constitutive sensitivity to CXCL12–CXCR4 axis signaling through G_αi2_ (33), MAGIS results in the opposite—a constitutive insensitivity to CXLC12–CXCR4 signaling (and to signaling for other G_αi2_-dependent GPCRs) due to G_αi2_-GPCR receptor decoupling (3). Thus, mechanistically MAGIS syndrome presents as a CXCR4 signaling deficiency. This is evidenced in the propensity of MAGIS patients to have increased circulating CXCR4 sensitive cell populations such as neutrophils, B cells, and monocytes (3).

**Figure 4.**
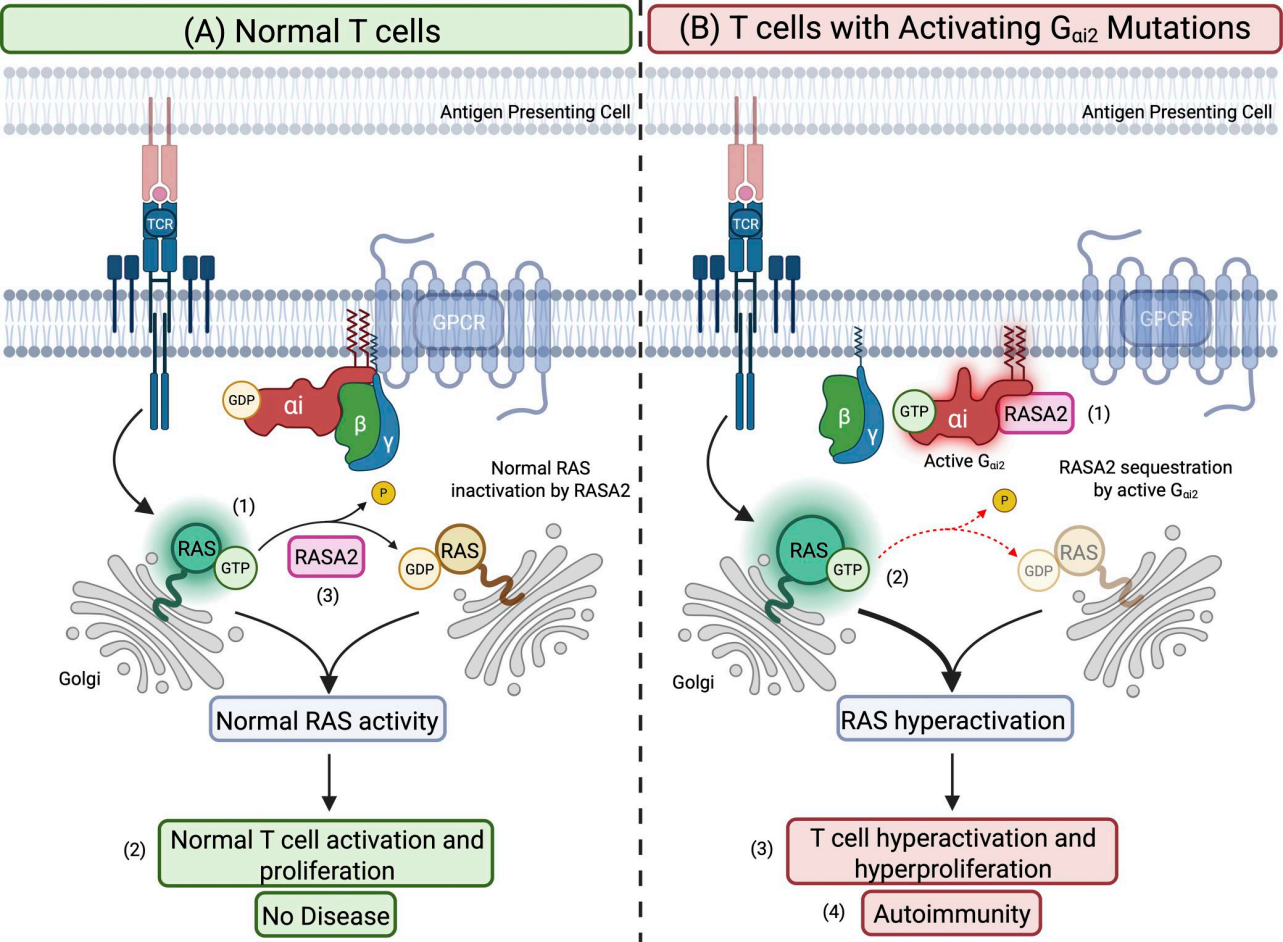
**Active G**_**αi2**_ **sequesters RASA2 to promote RAS activation and T cell hyperresponsiveness. (A)** In normal T cells, TCR stimulation leads to RAS activation (1), which drives T cell activation and proliferation (2). RASA2, a GAP for RAS, facilitates the hydrolysis of RAS-GTP into inactive RAS-GDP (3). In this way, RASA2 functions to inactivate RAS and limit normal T cell responses. **(B)** Activating mutations in G_αi2_ sequester RASA2 away from RAS in the Golgi apparatus (1). Since RASA2 normally accelerates the hydrolysis of RAS-GTP into the inactive RAS-GDP, this sequestration promotes TCR-induced activation of RAS (2) and downstream signaling required for T cell growth and proliferation. Consequently, in patients with activating mutations in G_αi2_, the stimulatory requirement for full T cell activation and proliferation is reduced by increased RAS activation. The enhanced TCR-induced activation and proliferation (3) may explain the autoimmunity (4) observed in some patients.

Beyond immunodeficiency, MAGIS patients also present with life-threatening autoimmune disease despite normal frequencies of regulatory T cells (CD4^+^FOXP3^+^CD25^high^) or autoreactive B cells (CD19^+^CD21^lo^CD38^lo^) in peripheral blood (3). Instead, under various TCR-stimulating conditions, T cells from MAGIS patients show enhanced activation and proliferation, and these phenotypes can be phenocopied in healthy donor primary T cells by expressing MAGIS mutant proteins (3). Upon TCR stimulation, the mutant G_αi2_ proteins do not impact proximal TCR signaling; rather, they promote enhanced RAS activation and downstream ERK/MAPK and PI3K/AKT/S6 signaling pathways required for cellular growth and proliferation (3). Utilizing quantitative proteomics, we identified RASA2, a GAP for RAS, as an effector target of G_αi2_, and found that active G_αi2_ inhibits RASA2-mediated negative regulation of S6-regulatory signaling and T cell activation (3). Instead of directly inhibiting RASA2’s GAP activity toward RAS, the activating mutant G_αi2_ sequesters RASA2 toward the plasma membrane (3). This spatial regulation of the RAS gatekeeper enhances TCR-induced RAS activity required for T cell activation and proliferation (Fig. 4). In MAGIS patients with autoimmunity, enhanced RAS activity and resulting T cell hyperresponsiveness may cause breakdowns in peripheral tolerance, predisposing to autoimmunity and age-associated lymphocytosis.

## Cyclic AMP (cAMP) and MAGIS

**Figure 5.**
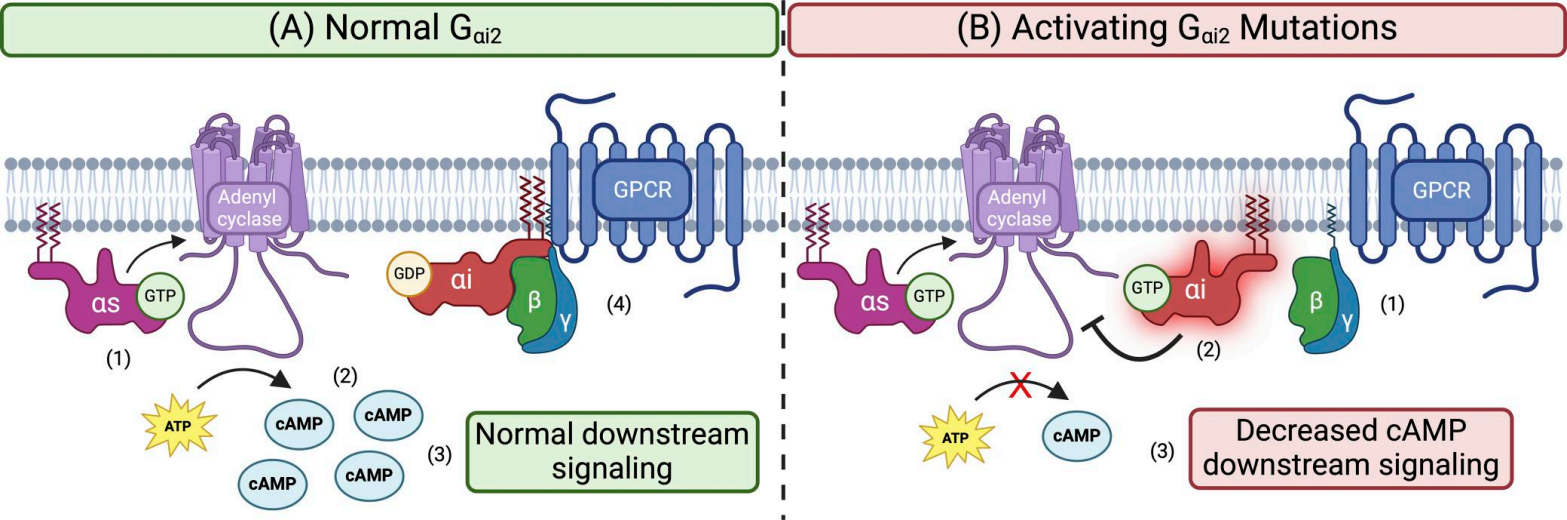
**Activating G**_**αi2**_ **mutations inhibit AC’s production of cAMP. (A)** Following GPCR stimulation, GTP-bound G_αs_ subunits promote (1), while GTP-bound G_αi_ subunits oppose (2), AC production of cAMP from ATP. The net balance of G_αs_ and the G_αi_ activities dictates the level of cAMP produced, which then functions as a second messenger to regulate various cellular responses (3). The extent of cAMP inhibition is limited by the normal cycling of WT G_αi2_ proteins into their inactive GDP-bound state (4). **(B)** Prolonged cycling of activating mutant G_αi2_ proteins in the patients’ cells disproportionately inhibits AC (1), thereby decreasing intracellular cAMP levels (2), which results in decreased downstream signaling (3). This may be responsible for other facets of the patients’ clinical features, such as impaired endocrine responses.

Inhibitory heterotrimeric G_α_ proteins (G_αi_) are named for their inhibitory effect on adenylyl cyclase (AC), the primary producer of cAMP (34). Indeed, the activating G_αi2_ variants in MAGIS inhibit cAMP production and reduce intracellular cAMP levels (3) (Fig. 5). cAMP is a ubiquitous second messenger and regulates a broad range of physiological processes including cell proliferation, migration, metabolism, and many others (35). cAMP is generated in response to extracellular stimuli, such as hormones and neurotransmitters, by the effect of stimulatory heterotrimeric G proteins (G_αs_) on AC (36). The production of cAMP is counterbalanced by G_αi/o_ proteins, which can inhibit activity of some AC isoforms (37, 38), and by phosphodiesterases (PDEs), which promote cAMP degradation (39). The cellular effect of cAMP is highly context-dependent, influenced by the expression of individual AC isoforms, PDE isoforms, downstream effectors, and unique receptor–ligand stimuli (35). Within T cells, pharmacological elevation of cAMP levels has been shown to inhibit both chemotaxis (40, 41) and TCR-induced activation and proliferation, the latter through a well-described effect on proximal TCR signaling (42, 43).

Given the central role of G_αi_ proteins in cAMP biology and the centrality of cAMP in myriad cellular responses including cell migration and T cell activation, we investigated the contribution of this pathway in MAGIS physiology. To simulate the low intracellular cAMP levels seen in MAGIS patient T cells due to G_αi_ activation, we ablated endogenous cAMP production in primary healthy donor T cells via CRISPR knockout of the major AC isoforms in T cells (AC3 and AC7) (3). Surprisingly, these manipulations did not impair T cell chemotaxis nor did they enhance T cell activation and proliferation as seen in MAGIS patient cells. These data suggest the impaired chemotaxis and T cell hyperresponsiveness of MAGIS syndrome are independent of cAMP and related to the mechanisms described above. As cAMP has many other immune functions including inhibition of proinflammatory cytokine production from dendritic cells and macrophages (44, 45, 46), cytotoxic function of natural killer cells (47), or antibody responses by B cells (46), further investigations are necessary to understand the role of altered cAMP levels in the immune and nonimmune features of MAGIS and may lead to cAMP-related treatment in this disease.

## Possible treatment modalities

Options for treating severe immune abnormalities in MAGIS may include hematopoietic stem cell transplantation (HSCT). Replacement with hematopoietic cell progenitors from a healthy donor could correct the defective leukocyte chemotaxis and T cell hyperresponsiveness in this disease. Results were encouraging for one MAGIS patient who received fully matched unrelated donor stem cells and achieved full donor chimerism (see Supplementary Text 4 in [3]). HSCT successfully eradicated the patient’s granulomatous skin disease caused by vaccine-strain rubella virus, as well as his recurrent upper respiratory tract infections, otitis media, and bronchitis. The patient also had a history of autoimmune hemolytic anemia, which has remained in remission over 3 years after HSCT although he receives immunosuppressant medications for chronic graft-versus-host disease. As expected, HSCT did not affect his nonimmune disease manifestations including autism spectrum disorder and growth hormone deficiency. One caveat is that HSCT has generally been comparatively less successful for autoimmune diseases than immunodeficiency (reviewed in [48]). Furthermore, HSCT for autoimmunity has primarily been done as autologous transplants, which would not likely work for MAGIS. Thus, caution must be taken in drawing conclusions from the limited experience to date.

Other possible treatment modalities were suggested from our initial study delineating pathogenic mechanisms of MAGIS. In our initial characterization of this disease, we were fortunate that one patient had a protospacer adjacent motif sequence in her genomic DNA that enabled Cas9/CRISPR-mediated selective deletion of the mutant but not WT *GNAI2* allele. This manipulation was able to correct the T cell hyperresponsiveness in vitro, although effects on chemotaxis were not tested (3). Furthermore, inhibitors of the RAS/ERK/MAPK and PI3K/AKT pathways were each able to partially correct the T cell hyperresponsiveness in vitro, although both inhibitors were required for complete correction (see Fig. S17 in [3]). These results suggest that other treatment options for MAGIS patients may include gene therapy or combinations of small molecule inhibitors of the downstream signaling pathways leading to ribosomal S6 protein activation in T cells, such as the mTOR inhibitor sirolimus. Alternatively, developing compounds that can more proximally target the abnormally increased G_αi2_-RASA2 interaction in MAGIS T cells may be a considered as a future potential therapeutic strategy for autoimmunity. It is worth noting that despite some phenotypic overlap and shared affected pathways between MAGIS and WHIM syndromes, WHIM-specific treatments such as plerixafor or mavorixafor are unlikely to be useful in MAGIS due to the distinct molecular mechanisms of these diseases (see the “Biochemical impact” section above) (49, 50).

Finally, it is possible that the decreased cAMP in MAGIS contributes to some of the disease features. While we did not demonstrate a role for cAMP in leukocyte migration or T cell hyperresponsiveness, the decreased cAMP may exert pathogenic effects in other cell types. For example, cAMP seems to suppress inflammation through its effects on myeloid cells (51), suggesting that decreased cAMP in those cell types might contribute to exaggerated inflammatory responses in some MAGIS patients. Small molecule inhibitors of PDE can raise intracellular cAMP by blocking the degradation of cAMP to AMP. Specific PDE4 inhibitors have been approved by regulatory agencies for the treatment of atopic dermatitis, psoriasis, psoriatic arthritis, asthma, or chronic obstructive pulmonary disease (52). As some of these conditions are observed in MAGIS patients, normalizing their cAMP levels by treating with PDE inhibitors might be considered. Furthermore, it is also possible that the decreased cAMP is responsible for abnormal functioning of the endocrine or other systems in MAGIS. If so, PDE inhibitors might have broader effects beyond the immune system in treating the patients. However, PDE inhibitors would not correct for abnormal functioning secondary to developmental birth defects.

## Conclusions

MAGIS patients can present heterogeneous clinical features, most often involving abnormalities of the immune, endocrine, skeletal, and nervous systems. Rare pathogenic activating *GNAI2* mutations responsible for disease can be recurrent and either de novo or transmitted in an autosomal dominant manner. The detailed clinical characterization of this disease should facilitate identification of other MAGIS patients. Elucidation of the molecular pathogenic mechanisms suggests several potential strategies for treating severely affected patients. In particular, future studies examining how the suppressed cAMP contributes to inflammatory and nonimmune disease in MAGIS patients should help clarify whether treating them with available drugs that increase cAMP levels will be beneficial.
